# The Impact of NLRP3 Activation on Hematopoietic Stem Cell Transplantation

**DOI:** 10.3390/ijms222111845

**Published:** 2021-10-31

**Authors:** J. Luis Espinoza, Kosuke Kamio, Vu Quang Lam, Akiyoshi Takami

**Affiliations:** 1 Faculty of Health Sciences, Kanazawa University, Kanazawa 920-0942, Japan; koooo.201@stu.kanazawa-u.ac.jp; 2 Division of Hematology, Department of Internal Medicine, Aichi Medical University School of Medicine, Nagakute 480-1195, Japan; quanglamvu1991@gmail.com (V.Q.L.); takami-knz@umin.ac.jp (A.T.); 3 Hematopoietic Cell Transplantation Center, Aichi Medical University Hospital, Nagakute 480-1195, Japan

**Keywords:** inflammasome, bone marrow transplantation, transplant related mortality, NLRP3 genotypes, transplant outcomes

## Abstract

NLR family pyrin domain-containing 3 (NLRP3) is an intracellular protein that after recognizing a broad spectrum of stressors, such as microbial motifs and endogenous danger signals, promotes the activation and release of the pro-inflammatory cytokines IL-1β and IL-18, thus playing an essential role in the innate immune response. Several blood cell types, including macrophages, dendritic cells, and hematopoietic stem and progenitor cells (HSPCs), express NLRP3, where it has been implicated in various physiological and pathological processes. For example, NLRP3 participates in the development and expansion of HSPCs, and their release from bone marrow into the peripheral blood has been implicated in certain hematological disorders including various types of leukemia. In addition, accumulating evidence indicates that activation of NLRP3 plays a pivotal role in the development of transplant complications in patients receiving hematopoietic stem cell transplantation (HSCT) including graft versus host disease, severe infections, and transplant-related mortality. The majority of these complications are triggered by the severe tissue damage derived from the conditioning regimens utilized in HSCT which, in turn, activates NLRP3 and, ultimately, promotes the release of proinflammatory cytokines such as IL-1β and IL-18. Here, we summarize the implications of NLRP3 in HSCT with an emphasis on the involvement of this inflammasome component in transplant complications.

## 1. Introduction

Hematopoietic stem cell transplantation (HSCT) involves the intravenous infusion of hematopoietic stem and progenitor cells (HSPCs) in order to reestablish blood cell production in patients whose bone marrow (BM) or immune system are damaged or defective due to the fact of a variety of acquired and inherited malignant and nonmalignant disorders [1,2].

According to the worldwide Network of Blood and Marrow Transplantation, every year, nearly one hundred thousand HSCTs are performed worldwide, and the number continues to increase by 10–20% annually (https://www.wbmt.org/; accessed date on 30 September 2021); however, despite considerable improvements in conditioning regimens, prevention and management of infection complications, and supportive care, HSCT remains a high-risk procedure that often associates with severe complications, such as severe infections, conditioning regimen-related tissue damage, veno-occlusive disease (VOD), and acute or chronic graft-versus-host disease (GvHD) and, therefore, this highly specialized therapy is reserved for patients with life-threatening diseases for whom there are no other therapeutic options available [3,4,5]. This includes hematologic malignancies, such as certain high-risk leukemias, malignant lymphomas, and multiple myeloma, where HSCT is performed to either eliminate malignant cells infiltrating the BM or to allow patients to receive high-dose chemotherapy. In addition, HSCT is utilized as a curative therapy for nonmalignant acquired BM failures, such as aplastic anemia, and in certain genetic diseases with abnormal or defective hematopoiesis such as thalassemia, sickle cell anemia, and severe combined immunodeficiency [2,4].

The immunosuppressive and myeloablative conditioning regimens (high-dose chemotherapy or irradiation given immediately prior to a transplant) utilized in HSCT, particularly in the case of allogeneic transplantation, induces widespread damage to the recipient’s normal tissues and is manifested by the disruption of various homeostatic mechanisms [1,2]. Moreover, patients receiving HSCT present a profound alteration of the composition and diversity of the commensal microbiome (dysbiosis) which, along with the mucosal barrier injury of the gastrointestinal tract associated with this procedure, promote bacterial translocation which, in turn, predisposes to the emergence of severe infections and acute GvHD. In addition, the inflammatory response triggered by HSCT induces the production and release of proinflammatory cytokines, leading to immune dysregulation [6,7]. Consequently, the severe tissue damage associated with HSCT often triggers a massive immune response that can result in life-threatening complications.

A critical player in the acute inflammatory response, such as that observed in the transplant setting, is the NLR family pyrin domain-containing 3 (NLRP3) inflammasome. This intracellular sensor detects a broad array of microbial motifs and other danger signals such as extracellular ATP and crystalline uric acid [8]. Upon its activation, NLRP3 triggers the maturation and release of interleukin (IL)-1β and IL-18, thus initiating or promoting inflammatory responses under physiological and pathological conditions.

NLRP3 is expressed by various cells of the hematopoietic system including monocytes, neutrophils, dendritic cells, and HSPCs [9]. NLRP3 is crucial in the host’s innate immune response, especially in combating pathogen infections; however, excessive NLRP3 activation in response to extensive tissue damage, such as that observed in patients receiving HSCT, plays a critical role in the development of transplant complications. In this article, we summarized the preclinical and clinical evidence on the participation of NLRP3 activation in HSCT with an emphasis on the role of excessive inflammasome activation in the development of transplant complications including GvHD, TRM, severe infections, and disease relapse.

## 2. NLRP3 in Hematopoiesis and Leukemogenesis

The inflammasome is a cytoplasmic multi-protein complex of the innate immune system that is activated in response to a variety of danger-associated signals including infection, metabolic stress, and any sort of tissue damage [10,11]. Various key inflammasome components (NLRP1, NLRP3, NLRC4, AIM2, NLRP6, NLRP7, and NLRP12) have been described so far, NLRP3 (also known as cryopyrin) being the most widely characterized. Mechanistically, inflammasome activation is initiated by various types of cytosolic pattern recognition receptors (PRRs). This includes the nucleotide-binding oligomerization domain and leucine-rich repeat-containing receptors (NLRs), absent in melanoma 2 (AIM2), IFN-inducible protein 16 (IFI16), and pyrin [11]. These PRRs respond to either microbe-derived pathogen-associated molecular patterns (PAMPs), such as bacterial LPS and viral dsRNA, or danger-associated molecular patterns (DAMPs) generated by the host cell (e.g., uric acid crystals, ATP, and reactive oxygen species). Upon activation, inflammasome leads to the processing and secretion of the inflammatory cytokines IL-1β and IL-18 and also triggers the cleavage of gasdermin-D, which promotes an inflammatory form of programmed cell death termed pyroptosis [12].

Appropriate NLRP3 inflammasome activation is crucial for the host to successfully eliminate foreign pathogens and to recover homeostasis after tissue damage; however, aberrant inflammasome activation can cause uncontrolled tissue responses that may contribute to various acquired inflammatory diseases, including systemic juvenile idiopathic arthritis, cancer, and certain neurodegenerative diseases [13].

In addition, inherited autoimmune diseases such as familial cold autoinflammatory syndrome (CAS), Muckle–Wells syndrome (MWS), and neonatal-onset multisystem inflammatory disease (NOMID). These disorders are caused by different mutations in the *NLRP3* gene, which cause excessive production of IL-1β and are responsive to treatment with anakinra, a selective antagonist of the IL-1β receptor [11].

Traditionally, NLRP3’s function has mainly been studied in professional immune cells of the innate immune system, particularly in monocytes/macrophages; however, accumulating evidence indicates inflammasome component expression in the epithelial barrier tissues, where they have been shown to represent an important first line of defense, and other immune cell types, such as dendritic cells, monocytes, and neutrophils, express high levels of NLRP3 [12,14]. Furthermore, HSPCs express NLRP3, where it has been implicated in physiological and pathological signals. For example, NLRP3 participates in the development and expansion of HSPCs and their release from bone marrow (BM) into the peripheral blood and has been implicated in certain hematological disorders including various types of leukemia [15]. In preclinical studies, NLRP3 inflammasome signaling promoted the expansion of HSPCs [12] and also played a pivotal role in the mobilization of HSPCs from BM into peripheral blood and was crucial for optimal homing, engraftment, and hematopoietic reconstitution, and this process can be drastically affected by the conditioning used in the setting of transplantation [16].

NLRP3 activation has been shown to interfere with hematopoiesis (the process of making new blood cells). This phenomenon is better delineated during aging, where an increase in the number of myeloid-biased HSPCs and myeloid cells leads to the dominance of myelopoiesis over lymphopoiesis which is, in part, due to the release of NLRP3 inflammasome-derived IL-1β, ultimately leading to a shrinkage of the pool of B and T lymphocytes over time [17]. In addition, during aging, the release of IL-1β and IL-18 from innate immunity cells inhibits erythroid colony formation, and the activation of IL-1 receptor signaling leads to a decrease in erythropoietin secretion in the kidney, ultimately leading to defective erythropoiesis [18], thereby impairing the production of erythrocytes.

A recent study showed that NLRP3 inflammasome promotes AML progression in an IL-1β-dependent manner. In patient-derived samples, NLRP3 was overexpressed and highly activated in AML bone marrow leukemia cells, and this correlated with poor prognosis, and the activation of NLRP3 in AML cells promoted leukemia cell proliferation, inhibited apoptosis, and increased resistance to chemotherapy [19].

Similarly, in a mouse model of leukemia, animals under chronic stress exhibited significantly increased tissue infiltration of leukemic cells and impaired survival, which was accompanied by elevated NLRP3 expression and higher levels of IL-1β in the liver or bone marrow and secreted IL-1β in the plasma [20]. On the other hand, immune cell activation via NLRP3 was recently shown to promote antileukemic activities. NLRP3-activated BM dendritic cells induced IL1β-dependent immunity and promoted the differentiation of CD4+ T cells into tumor-specific interferon-γ (IFN-γ)-producing T helper 1 (Th1) cells recognizing and eliminating leukemia cells [21]; this suggest that NLRP3-activated immune cells, namely, dendritic cells and Th1 cells, may have a therapeutic immunotherapeutic potential in AML.

Together, these data suggest that NLRP3 inflammasome is implicated in the maintenance of normal homeostasis of the hematopoietic system, playing important roles in the homing and expansion of HSPCs and immune response to pathogens; however, dysregulated NLRP3 inflammasome is implicated in various pathogenic processes such as leukemia progression and the development and severity of GvHD.

## 3. Role of NLRP3 in TRM and Infection Complications after HSCT

Conditioning, which implies the administration of high-dose chemotherapy or irradiation immediately prior to a transplant, is an essential component of HSCT and is required for supporting the engraftment of donor HSPCs without rejection by the recipient’s immune system, and it plays a pivotal role in eradicating malignant cells; however, conditioning induces profound damage in the recipient’s tissues leading to end-organ damage. In addition, tissue damage associated with the profound immunosuppressive status of patients undergoing HSCT increases exponentially the risk of developing severe infections; thus, infection complications are among the major causes of morbidity and mortality in HSCT recipients [6,22]. Infections are commonly caused by microorganisms derived from the gastrointestinal tract or by opportunistic organisms colonizing the skin that enter the bloodstream via intravenous catheters [6,22].

The engraftment of donors cells occurs in a period ranging from six to 84 days after HSCT, and this process is crucial for the reconstitution of the host’s immune system and, hence, to combat microbial infections [1]. There are three well-defined phases of immune reconstitution after HSCT, and specific microbial infections predominate in each phase (i.e., pre-engraftment, early post-engraftment, and late post-engraftment phase). Thus, during the early pre-engraftment phase (<30 days after HSCT), bacterial infections, particularly those due to the presence of Gram-positive organisms, are predominant.

Other pathogens implicated in this phase include *Clostridioides difficile*, fungi (*Candida* spp. and *Aspergillus* spp.), and herpes simplex virus (HSV) [23]. During the early post-engraftment phase (30–100 days after HSCT), Gram-positive bacteria also predominate over Gram-negative bacteria [24,25], which often manifest as pneumonia and gastrointestinal infections. In the late post-engraftment phase (>100 days after HSCT), infections caused by encapsulated bacteria along with invasive fungal infections are frequently observed. In addition, reactivation of viral infections, especially cytomegalovirus (CMV), varicella–zoster virus (VZV), and Epstein–Barr virus (EBV) can be seen during this period [1,22].

Viral infections, especially those caused by cytomegalovirus, influenza A virus, and adenovirus [26], which constitute common causes of infections in patients undergoing HSCT, generate a variety of PAMPs and DAMPs that are recognized by NLRP3 inflammasome, the activation of which triggers the proteolytic processing of gasdermin D, which causes pore formation in the membrane of infected cells leading to pyroptosis cell death [27]. In addition, the secretion of IL-1β subsequently recruits immune cells, such as neutrophils and NK cells, to the site of infection, which also contributes to the elimination of invading viruses. Moreover, both NLRP3-dependent cytokines (IL-1β and IL-18) contribute to the activation of the adaptive immune response to further potentiate the host’s antiviral responses [28].

NLRP3 inflammasome is particularly important in the host’s response to several bacterial pathogens, such as IL-1β and IL-18, released upon NLRP3 activation, contributing to the enhancement of the immune response against invading bacteria. Diverse mechanisms by which pathogenic bacteria activate NLRP3 inflammasome have been identified, the bacterial release of pore-forming toxins being one of the best characterized. For example, *Staphylococcus aureus* activates the NLRP3 inflammasome by releasing bacterial lipopeptides, which activates Toll-like receptor 2 (TLR2) and by directly releasing the pore-forming toxin α-hemolysin. Similarly, pneumolysin, secreted by *Streptococcus pneumoniae* [29], and *Clostridioides difficile* toxins A and B [30] are known to activate NLRP3. Listeria monocytogenes, another pathogen causative of severe infections in transplant recipients and other immunocompromised individuals, induces NLRP3 activation via phagosomal membrane damage by secreting the toxin listeriolysin O [31].

Direct activation of NLRP3 inflammasome has also been documented by pathogenic fungal products derived from *Candida albicans* and *Aspergillus* [32,33], and infections with these pathogens are an important cause of morbidity and mortality among HSCT recipients [1]. Both IL-1β and IL-18 have been shown to exert protective effects against various fungal infections. For example, mice deficient in IL-1α or IL-1β are more susceptible to systemic *Candida albicans* infection, which is in part due to the critical role of IL-1 in neutrophil recruitment [34]. Fungal recognition via Dectin-1, a recently characterized PRR, facilitates IL-18 production which, in turn, contributes to the generation of protective antibodies against *Candida albicans* [35]. Moreover, patients with active paracoccidioidomycosis presenting with high levels of IL-1β and IL-18 in the serum that return to normal levels in response to antifungal therapy indicate that these cytokines are released during active infection with this pathogen [36]. Interestingly, it appears that during fungal infections involving mucosal tissues, interleukin-1 receptor (IL-1R) along with IL-36R are required for neutrophil recruitment to the site of infection, and in the case of fungal systemic disease, IL-18 mediates protective functions by triggering Th1 responses [37]. Together, the above observations indicate that NLRP3-induced cytokines play a key role in mediating immunity against different fungal infections.

However, dysregulated NLRP3 inflammasome activation in response to the above-mentioned pathogens can also lead to severe tissue damage and systemic inflammatory responses. The aberrant recruitment and activation of inflammatory cells at the sites of infection along with the pyroptosis-mediated cell death have been shown to aggravate the disease severity [38].

Uric acid is an endogenous activator of NLRP3 and upon its interaction with the inflammasome system, it triggers danger signals that enhances T-cell responses. Data from preclinical studies indicate that GvHD is aggravated by uric acid via NLRP3 inflammasome-mediated IL-1 production [39]. Interestingly, high uric acid levels before the start of transplant conditioning correlated with increased mortality after allogeneic HSCT, where patients with increased levels of uric acid had significantly shorter overall survival (HR 2.8, 95% CI: 1.7–4.7, <0.0001) and significantly increased non-relapse mortality after allogeneic HSCT recipients (HR 2.7, 95% CI: 1.4–5.0, =0.003). Similarly, the incidence of relapse after allogeneic HSCT increased in patients with higher uric acid levels (HR 1.6, 95% CI: 1.0–2.5, =0.04) [40]. Consistent with these observations, a pilot study reported reduced incidence of acute GvHD in patients undergoing allogeneic HSCT after depletion of uric acid by administering urate oxidase during myeloablative conditioning [41].

## 4. Role of NLRP3 in the Development of GvHD

Epidemiological and clinical studies have identified an array of risk factors, on both the donor and recipient sides, that determine transplant outcomes. For example, while patient age, presence of comorbidities, performance status, cytomegalovirus (CMV) status, and the patient’s disease (diagnosis, stage, and cytogenetic risk) are factors on the recipient side that predict outcome, donor characteristics that affect outcomes after transplant include gender, relation with the recipient, and age [42]. Similarly, genetic factors implicated in transplant outcomes have been also identified, with the level of human leukocyte antigen (HLA) mismatch between the donor and recipient being the most important genetic determinant of transplant outcomes. HLA genes are encoded in a segment on human chromosome 6p21.3 (the most variable region in the human genome) which has products that are involved in antigen presentation to CD8+ T cells, natural killer cells (NK cells), and CD4+ T cells and are crucial for enabling the immune system to recognize “self” versus “non-self” antigens [43]. Furthermore, using gene association studies, we and others have identified genetic variants in genes located outside the HLA region (non-HLA variants), especially single nucleotide polymorphisms (SNPs) in genes which have products that are associated with the immune response implicated in transplant outcomes [44,45,46,47,48].

GvHD is the most serious complication of allogeneic HSCT and occurs when donor-derived immune cells (mainly T-lymphocytes) recognize and attack histocompatibility antigens expressed on tissues of the transplantation recipient. Acute GvHD typically occurs within 100 days following HSCT, and the inflammatory response is present exclusively in three organs: the skin, the liver, and the gastrointestinal tract with defined stages according to extension and severity. On the other hand, chronic GvHD occurs beyond day 100 and generally affects the skin, gastrointestinal tract, and liver and is a major source of late treatment-related complications, although it less often results in death [49]. In addition, chronic GvHD may lead to the development of fibrosis, which may cause functional disability and require prolonged immunosuppressive therapy. Although GvHD has been historically classified into acute and chronic based on the timing of presentation with a cutoff of 100 days post-transplant, current evidence indicates that acute GvHD can occur even after day 100 (termed late acute GvHD) or some patients may present with an overlapping syndrome of both acute GvHD and chronic GvHD. Currently, the most accurate guidelines for the assessment and staging of acute GvHD are the Mount Sinai Acute GvHD International Consortium (MAGIC) criteria and the NIH 2014 criteria for chronic GvHD [49], the description of which is beyond the scope of this article. Mechanistically, GvHD is mediated by donor-derived immune cells that react to specific peptides of the host; thus, the presence of HLA allele mismatch between donor and recipient, especially HLA-B mismatch and multiple allele mismatches, associates with increased risks of acute GvHD and lower overall survival [50,51].

Data from preclinical studies indicate that NLRP3 activation plays a pivotal role in the development of acute GvHD. The excessive tissue damage induced by conditioning therapy leads to NLRP3 inflammasome activation that promotes IL-1β and IL-18 production, which prime immune cells implicated in the inflammatory response typical of GvHD [39]. In addition, inflammasome activation impairs the immune suppressive function of myeloid-derived suppressor cells, thus exacerbating GvHD [52]. In a mouse model of HSCT busulfan and cyclophosphamide (often used in transplant conditioning regimens) induced liver inflammation through NLRP3 activation, and the pharmacological inhibition of NLRP3 reduced infiltration of macrophages and neutrophils and improved liver function [53], thus suggesting that targeting NLRP3 may have a therapeutic potential to prevent transplant complications, particularly in the prophylaxis of liver inflammation after HSCT. Moreover, inflammation and pyroptosis triggered by NLRP3 inflammasome activation play a crucial role in aggravating oxidative stress and endothelial dysfunction [13], and accumulating evidence indicates that endothelial dysfunction is involved in various complications of HSCT such as VOD, transplant-associated thrombotic microangiopathy, and refractory acute GvHD [54].

## 5. NLRP3 and Disease Relapse

Disease recurrence or relapse after HSCT is a devastating event that is usually associated with a poor prognosis; however, a subset of patients can achieve a disease remission after a second allogeneic HSCT or may respond to further treatments. In general, relapse results from residual malignant cells that evade the toxicity of condition regimens, survive extensive chemotherapy, and escape the graft-vs-leukemia mediated by donor T cells [55]. Disease relapse after HSCT implies that malignant cells are highly resistant to the immune response mediated by donor lymphocytes. Resistance to immune cells can be mediated by several mechanisms, including the secretion of immunosuppressive cytokines, such as IL-10 and transforming growth factor beta (TGF-β) [56,57], and the upregulation of immune checkpoint inhibitors, such as programmed death-ligand 1 and 2 (PD-L1 and PDL2), and cytotoxic T-lymphocyte-associated protein (CTL-4) [58], where at least, in part, NLRP3 activation appears to be implicated. For example, data from patients with diffuse large B-cell lymphoma (DLBCL) have shown that NLRP3 activation upregulated PD-L1 and reduced the proportion of cytotoxic T cells [59]. This is supported by preclinical studies showing that NLRP3 activation contributes to the recruitment of granulocytic myeloid-derived suppressor cells into tumor tissues, thereby dampening the resulting antitumor immune response, and the pharmacologic inhibition of NLRP3 blocked the immune suppressive effects of granulocytic myeloid-derived suppressor cells and significantly augmented the efficacy of anti–PD-1 antibody immunotherapy [60].

Another study found that a high expression of caspase 1 and NLRP3 was associated with glucocorticoid resistance in acute lymphoid leukemia (ALL) thereby resulting in a poor prognosis. Mechanistically, the study showed that caspase1/NLRP3 overexpression induces glucocorticoid resistance via caspase cleavage of the glucocorticoid receptor in its transactivation domain, reducing cellular levels of functional glucocorticoid receptor and diminishing glucocorticoid transcriptional effects [61]. Another important finding of this study was the higher expression of caspase 1 and NLRP3 in leukemia cells obtained at the time of disease relapse [61], which is consistent with other studies reporting that leukemia cells at relapse are more resistant to prednisolone and dexamethasone compared to leukemia cells at initial diagnosis [62]. Given the critical role of NLRP3 in promoting inflammation, these results raise the possibility that during inflammatory processes, caspase 1 negatively regulates anti-inflammatory glucocorticoid signaling to further amplify the pro-inflammatory effects of NLRP3 inflammasome.

## 6. The Effects in Polymorphisms in *NLRP3* Expression and Function

The human *NLRP3* gene is located on chromosome 1q44, and nearly 60 SNPs within the entire *NLRP3* gene have been identified so far. Gene association studies have linked some of these SNPs, especially *rs10754558 (29940G>C), rs35829419* (Q705K)*, rs10925019, rs4925648,* and *rs4612666*, with susceptibility to various inflammatory conditions, including rheumatoid arthritis, ulcerative colitis, and others [63,64,65]. In particular, the variant *rs10754558* (29940 G>C), located in the 3′-untranslated region (3′-UTR) of the *NLRP3* gene has been shown to regulate NLRP3 expression [66] and has been associated with increased susceptibility to several inflammatory conditions [67], and a recent meta-analysis of eight studies (1764 cases and 1661 controls) found a significant association of the G allele of *NLRP3 rs10754558* with type 1 diabetes, rheumatoid arthritis, and systemic lupus erythematosus [68].

The fact that genetic variants in non-HLA genes can predict transplant outcomes has prompted various research groups to investigate the potential involvement of *NLRP3* polymorphisms in clinical outcomes after HSCT. One of the first studies to investigate the impact of genetic variants in the NLRP3 gene on transplant outcomes analyzed a small cohort of 133 HLA-identical sibling pairs undergoing allogeneic HSCT and observed that donor TT genotype at *rs10925027* in *NLRP3* was associated with disease relapse (odds ratio (OR) = 6.3, P = 1 × 10^–7^), thus suggesting a potential prognostic value of NLRP3 inflammasome genetic variants in allogeneic HSCT [69].

**Figure 1:**
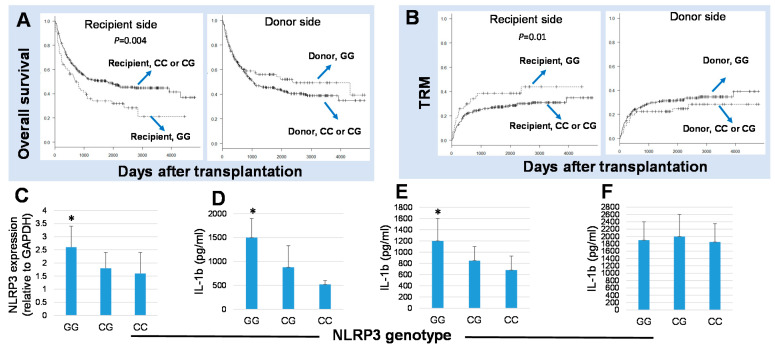
NLR family pyrin domain-containing 3 (NLRP3) genotypes influenced transplant outcomes after HSCT and also influenced IL-1β secretion in response to NLRP3 activation. The Kaplan–Meier analysis of the 5-year overall survival (OS) (**A**) and the transplant-related mortality rates (TRM) (**B**) after transplantation according to the recipient and donor NLRP3 genotypes. Recipients with the G/G genotype of NLRP3 at r*s10754558* had the worst 5-year overall survival compared to those with the C/C or C/G genotypes (hazard ratio (HR), 1.86; 95% confidence interval (CI), 1.22–2.22; *p* = 0.004; higher TRM, HR, 2.28; 95% CI, 1.20–4.35; *p* = 0.01). (**C**) RNA extracted from peripheral blood mononuclear cells (PBMCs) of 24 healthy individuals were subjected to RT-PCR with TaqMan primers specific for the *NLRP3* gene, and the analysis showed a significantly higher NLRP3 transcripts in individuals harboring the G/G genotypes in comparison to those with the C/C or C/G genotypes. Blood samples from 27 healthy individuals with the GG (*n* = 8), CG (*n* = 14), and CC (*n* = 5) genotypes of *rs10754558 NLRP3* were subjected to a whole blood culture assay for 16 h in the presence or the absence of LPS (**D**), the NLRP3 ligand MDP (**E**), or PHA (**F**), and the levels of IL-1β released in the culture supernatant were determined by ELISA. * *p <* 0.05.

Our group analyzed the impact of *rs10754558* genotypes in a cohort of 392 pairs of patients with hematologic malignancies and their HLA-unrelated 12/12 matched bone marrow donors transplanted through the Japan Donor Marrow Program (JDMP) and observed that recipients harboring the risk genotype G/G of *rs10754558* had a significantly worse five-year survival (hazard ratio (HR), 1.86; 95% confidence interval (CI), 1.22 to 2.22; *p* = 0.004) and higher TRM (HR, 2.28; 95% CI, 1.20 to 4.35; *p* = 0.01). No transplant outcomes were associated with the donor NLRP3 genotypes (Figure 1A,B) [70]. In line with these observations, PBMCs from individuals with the *NLRP3* G/G genotype expressed significantly higher levels of NLRP3 transcripts than those with the G/C or C/C genotypes (Figure 1C). Experiments using whole blood cell cultures [71] from healthy donors stimulated with LPS, which stimulates NLRP3 via TLR2, showed that donors harboring the NLRP3 G/G genotype secreted significantly higher levels of IL-1β compared to donors with the G/C or C/C genotypes (Figure 1D). This is also the case when whole blood cells are stimulated with muramyl dipeptide (MDP), a known ligand of NLRP3 [72] (Figure 1E); however, no differences in IL-1β levels among NLRP3 genotypes were observed when whole blood cells were stimulated with phytohemagglutinin (PHA), a naturally occurring mitogen that activates various types of immune cells, including monocytes, NK cells, and lymphocytes, and which does not signal via NLRP3 inflammasome (Figure 1F) [70].

In another study also conducted in patients transplanted through the JMDP, SNPs in the NLRP3 gene along with HLA mismatch were associated with acute and extensive GVHD in Japanese individuals who underwent HSCT for hematological malignancies, although those genetic variants did not correlate with survival outcomes, including TRM and overall survival [73], which was likely influenced by the considerable proportion of donor/recipient HLA mismatch in the cohort of patients included in this study. In addition, in this study, the authors analyzed other SNPs in the *NLRP3* gene (i.e., *rs4612666* and *rs10925027*) and not the SNP (i.e., *rs10754558*) analyzed in our study. However, our group also found an association between the *rs10754558* and increased risk of acute GvHD in a different cohort composed of 659 recipients hematologic malignancies receiving unrelated fully matched (HLA 12/12) HSCT [74], thus indicating that NLRP3 variants are associated with susceptibility to transplant complications. Taken together these results suggest that, in particular, the SNP *rs10754558* is a variant that affects NLRP3 expression and is implicated in the secretion of IL-1β secondary to NLRP3 activation.

**Figure 2:**
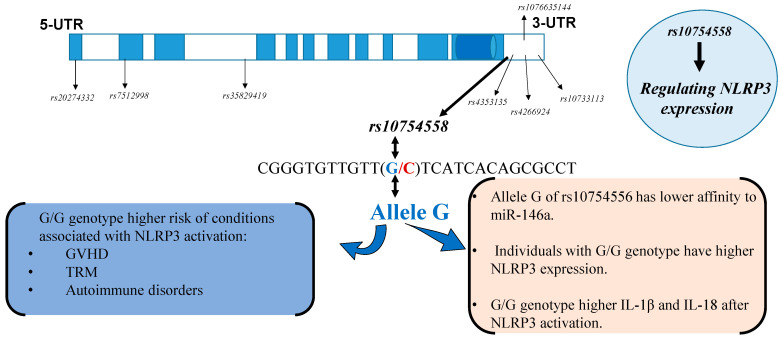
Schematic representation of *rs10754558* and other polymorphisms in the *NLRP3* gene with reported associations with susceptibility to various inflammatory diseases. Introns of the NLRP3 gene are displayed as blue boxes and exons are displayed as white boxes (not to scale). The SNP *rs10754558* is the 3′-untranslated region (3′-UTR) of the *NLRP3* gene and regulates gene expression. The G allele is a gain of function that decreases the affinity of NLRP3 to negative regulators (the microRNA, miR146a) and, consequently, individuals possessing the G allele have higher NLRP3 expression and secrete higher levels of cytokines in response to NLRP3 activation.

Interestingly, a recent study showed that the G/G genotype of *rs10754558* was significantly associated with increased risk of developing septic shock compared to the controls, and mechanistic studies utilizing luciferase reporter vectors containing NLRP3 3′-UTR with the *rs10754558* variation showed that the allele C had a higher affinity to miR-146a, a negative regulator of NLRP3 transcripts and, consequently, the allele C of *rs10754558* was more sensitive to the suppressive effects of miR-146a than the allele G [75]. These results further validated the functional relevance of *rs10754558* and established the molecular bases by which a genetic variant in the 3′-UTR of the *NLRP3* gene regulates NLRP3 expression, where a gain-of-function alteration (allele G) augments the resistance of NLRP3 transcripts to the suppressive regulatory effects of miR-146a-5p (Figure 2).

## 7. Concluding Remarks and Future Directions

The NLRP3 inflammasome is an important component of the innate immune system that recognizes an array of endogenous and invading pathogens driving protective inflammatory responses in the host. It also contributes to the expansion and migrations of HSPCs and appears to contribute to the maintenance of the hematopoietic pool. However, dysregulated NLRP3 activation, either due to the fact of rare mutations in the *NLRP3* gene or due to the fact of sequence variations in the *NLRP3* gene, has been implicated in the pathogenesis or the modulation of various inflammatory disorders. Whereas the crucial functions of NLRP3 inflammasome in the immune system and its role in the pathogenesis of various inflammatory disorders has been fairly characterized, its influence in hematological disorders have remained unknown until recently, when the implications of NLRP3 inflammasome in leukemia growth and progression and the development of GvHD is being deciphered.

Patients undergoing HSCT present a profound disruption in microbiome composition as a result of conditioning regimens, immunosuppressive therapy, and exposure to multiple antibiotics, and it is well known that intestinal dysbiosis plays a pivotal role in the development of transplant complications including GvHD, severe infections, and TRM [1,6,76]. Data from preclinical studies indicate that severe intestinal dysbiosis leads to the activation of the NLRP3 inflammasome, which can aggravate the inflammatory response to tissue injury [77,78]. It is currently unknown if such phenomenon (hyperactivation of NLRP3 in association with intestinal dysbiosis) also occurs in patients undergoing HSCT and, thus, further studies are needed to delineate the participation of NLRP3 in the development of transplant complications.

The involvement of the NLRP3 inflammasome in several inflammatory conditions has made it an attractive potential pharmacological target and, thus, the therapeutic utility of blocking the IL-1 pathway by using, for example, anakinra, the recombinant inhibitor of human IL-1R, which was approved for the treatment of certain autoimmune disorders, has been tested in various hematological conditions with variable results [76,79]. For example, whereas anakinra was effective in attenuating acute GvHD in preclinical models [80], in clinical trials, the blockade of IL-1 failed to prevent acute GvHD [81]. This contrasts with the efficacy of this therapy in ameliorating intestinal mucositis, cancer cachexia, and anthracycline-induced cardiotoxicity [82], which may suggest that the efficacy of IL-1 inhibition in humans may be influenced by timing, dosing, and the specific context and by the dual roles of NLRP3 inflammasome in these disorders.
